# Minimum data set harmonization in the management of cross-border Multi Casualty Incidents. Modified Delphi (VALKYRIES—H2020 project)

**DOI:** 10.1371/journal.pone.0305699

**Published:** 2024-07-18

**Authors:** Navid Behzadi Koochani, Raúl Muñoz Romo, Ignacio Hernández Palencia, Sergio López Bernal, Carmen Martin Curto, José Cabezas Rodríguez, Almudena Castaño Reguillo

**Affiliations:** 1 Servicio de Urgencias Médicas de la Comunidad de Madrid (SUMMA112), Madrid, Spain; 2 Fundación para la Investigación e Innovación Biosanitarias en Atención Primaria (FIIBAP), Madrid, Spain; 3 Facultad HM de Ciencias de la Salud de la Universidad Camilo José Cela, Spain; 4 Instituto de Investigación Sanitaria HM Hospitales, Spain; 5 Novotec Consultores S.A., Madrid, Spain; 6 University of Murcia, Murcia, Spain; 7 Tassica Emergency Training & Research, Spain; University Hospital RWTH Aachen & City of Aachen, GERMANY

## Abstract

**Introduction:**

There is a need to develop harmonized procedures and a Minimum Data Set (MDS) for cross-border Multi Casualty Incidents (MCI) in medical emergency scenarios to ensure appropriate management of such incidents, regardless of place, language and internal processes of the institutions involved. That information should be capable of real-time communication to the command-and-control chain. It is crucial that the models adopted are interoperable between countries so that the rights of patients to cross-border healthcare are fully respected.

**Objective:**

To optimize management of cross-border Multi Casualty Incidents through a Minimum Data Set collected and communicated in real time to the chain of command and control for each incident. To determine the degree of agreement among experts.

**Method:**

We used the modified Delphi method supplemented with the Utstein technique to reach consensus among experts. In the first phase, the minimum requirements of the project, the profile of the experts who were to participate, the basic requirements of each variable chosen and the way of collecting the data were defined by providing bibliography on the subject. In the second phase, the preliminary variables were grouped into 6 clusters, the objectives, the characteristics of the variables and the logistics of the work were approved. Several meetings were held to reach a consensus to choose the MDS variables using a Modified Delphi technique. Each expert had to score each variable from 1 to 10. Non-voting variables were eliminated, and the round of voting ended. In the third phase, the Utstein Style was applied to discuss each group of variables and choose the ones with the highest consensus. After several rounds of discussion, it was agreed to eliminate the variables with a score of less than 5 points. In phase four, the researchers submitted the variables to the external experts for final assessment and validation before their use in the simulations. Data were analysed with SPSS Statistics (IBM, version 2) software.

**Results:**

Six data entities with 31 sub-entities were defined, generating 127 items representing the final MDS regarded as essential for incident management. The level of consensus for the choice of items was very high and was highest for the category ‘Incident’ with an overall kappa of 0.7401 (95% CI 0.1265–0.5812, p 0.000), a good level of consensus in the Landis and Koch model. The items with the greatest degree of consensus at ten were those relating to location, type of incident, date, time and identification of the incident. All items met the criteria set, such as digital collection and real-time transmission to the chain of command and control.

**Conclusions:**

This study documents the development of a MDS through consensus with a high degree of agreement among a group of experts of different nationalities working in different fields. All items in the MDS were digitally collected and forwarded in real time to the chain of command and control. This tool has demonstrated its validity in four large cross-border simulations involving more than eight countries and their emergency services.

## Introduction

The Committee on Disaster Risk Reduction (UNDRR, UN Office for Disaster Risk Reduction) has defined a disaster as a serious disruption of the functioning of a community or society involving widespread human, material, economic or environmental losses and impacts, which exceeds the ability of the affected community or society to cope using its own resources [1] On that basis, it is noteworthy that disasters that occur in transborder areas are especially complex, especially when international intervention is required [2, 3]. In addition to the complexities of multi-victim incidents, cross-border events require the collaboration of different emergency providers, of different nationalities, speaking different languages, with different skills (emergency vehicles with medical staff are not always provided) [4] and with first responder systems that differ from one another (sometimes police or firefighters, even the military are the first on the scene) [5] in addition to the differences related to the prevention and promotion programmes of the different countries, the health systems they have (public and/or private) and the implication for the health of the patients attended [6], which is related to survival in the event of an event of these characteristics. Including the risk associated with the safety of staff working in this type of event.

Here we must acknowledge the fundamental role of the emergency medical services (EMS) in disaster situations with multiple casualties, when the lack of resources identified in the UN’s definition is particularly apparent along with the need for collaborative working across differing action protocols, means of communication and even laws. Such scenarios raise significant ethical issues, because critical decisions have to be taken quickly by responders, who may also become direct or indirect casualties in the course of their efforts.

It is in these scenarios that a highly effective chain of command and control translates into highly effective use of resources, the shortest time to intervention and, finally, the saving of human lives. There is a need to develop harmonized procedures and a Minimum Data Set (MDS) for cross-border Multi Casualty Incidents (MCI) to ensure appropriate management of such incidents, regardless of place, language and internal processes of the institutions involved. Such transnational incident management will be possible and have a significant social impact if there is prior agreement as to the information required, making use of the opportunities offered in terms of technology, procedures, collaboration, and training, by the Security Market of the European Union (EU), thus far very fractured.

Today there are already MDS as tools for the systematic collection of minimal basic information about episodes of care for individual patients to have a system of high-quality exhaustive and consistent information that allows improved process management [7, 8]. Thus, various works have validated MDS and so highlighted the need for consensus on the critical information required to support the management of disaster scenarios [9, 10].

Although great progress is being made in standardising processes and data collection through MDS, there are important differences not only between countries, but also between regions within the same country, especially in lower-middle-income countries [11]. There are also inequalities in terms of resources used, with large differences between hospitals, primary care centres or nursing homes, even within the same cities [12]. This is why, in 2015, the UN developed a document to help build and improve health electronic systems through the MDSs to improve data interoperability at national and subnational levels [13].

It is crucial to adopt clinical information structures that allow interoperability among the EU’s Member States under laws concerning patients’ rights in cross-border healthcare (Directive (EU) 2011/24 of the European Parliament and of the Council of 9 March 2011) [14]. This advance will also facilitate the availability of high-quality normalized data that may be useful in the context of the European Health Data Space [15], which is a health-specific ecosystem comprised of rules, common standards and practices, infrastructures and a governance framework that aims at empowering individuals through increased digital access to and control of their electronic personal health data, at the national level and EU-wide, and support to their free movement, as well as fostering a genuine single market for electronic health record systems, relevant medical devices and high-risk AI systems, providing a consistent, trustworthy and efficient set-up for the use of health data for research, innovation, policy-making and regulatory activities [16].

The EU has developed various tools for collective collaboration and cooperation between Member States and across industries. Of particular note is the EU’s Mandate M/487 [17], adopted to develop work plans and roadmaps for the normalization of standards in security and the protection of critical infrastructure in the EU. Mandate M/487 requires the identification of the challenges to be met in the use of terminology, planning, resilience and institutional interoperability.

In response to that need and in order to identify a set of solutions to improve collective resilience in the face of MCI, Project VALKYRIES was launched under the EU’s Horizon 2020 Framework Programme (Harmonization and Pre-Standardization of Equipment, Training and Tactical Coordinated procedures for First Aid Vehicles deployment on European MCI. Project VALKYRIES falls under the programme entitled ‘Secure Societies—Protecting freedom and security of Europe and its citizens’ and more specifically under the sub-programme ‘Increase Europe’s resilience to crises and disasters’, Sub-topic 3 ‘First Aid vehicles deployment, training, maintenance, logistic and remote centralized coordination means’, under procurement process SU-DRS03-2018-2019-2020, ‘Pre-normative research and demonstration for disaster-resilient societies’. This project was active from October 2021 to October 2023 a comprised a set of partners from a wide variety of EU countries and different expertise.

In that context and in light of the salient issues identified in EU M/487 [18] in terms of the gaps to be bridged in interoperability, incident management, organizational interoperability, and operational effectiveness, H2020-VALKYRIES identified the following objectives inspired by the Observe-Orient-Decide-Act loop (Boyd’s OODA Loop [19]).

### General objective

To optimize management of cross-border Multi Casualty Incidents through a Minimum Data Set collected and sent in real time to the chain of command and control for an incident.

### Specific objectives

1. To develop and agree a Minimum Data Set to ensure optimal management of cross-border Multi Casualty Incidents consistent with the processes, protocols and laws of the countries involved.
2. To determine the degree of agreement among experts as to the variables agreed to make up the Minimum Data Set.

## Materials and methods

To that end, a group of 6 researchers from the VALKYRIES project carried out a consensus data harmonization process to determine an MDS that could be collected and communicated electronically in real time to the chain of command and control, seeking optimal management of an MCI in cross-border areas regardless of the particular procedures, protocols, languages and location of the event.

That exercise was carried out through a combination of a variant of the Delphi method (modified Delphi) [20, 21] as a validated consensus tool and the Utstein technique or style [22] to select the essential data, as described above.

The basic principles that guided us in the selection of the final items were: the feasibility of collecting the data, real-time onward communication of the data to the whole chain of command and control and being thought to be essential to good management of an MCI.

Modified Delphi is an abbreviated version of the classic Delphi modified to the specific needs and resources of the project. Subsequently, abbreviated Delphi was complemented by the Utstein method to improve the quality of the data gathered and allow more precise comparison of results across different studies concerning emergency response.

For this purpose, the researchers were supported by 17 experts (with more than 20 years of experience in their field of expertise) from 17 agencies and institutions of 8 different countries, involved in the Valkyries project.

The MDS was presented and validated by external experts of the Valkyries project before being tested in the 4 major cross-border simulations foreseen in the project. The list and profile of researchers, internal and external experts can be seen in Table 1.

**Table 1:** Study participants classified by area of work and country of activity.

Organization name	Country	Profile
**List of researchers**		
*Servicio Madrileño de Salud (SERMAS)*	Spain	Emergency coordination
*Tassica emergency training & research S*.*A*. *(TASSICA)*	Spain	Emergency coordination
*University of Murcia (UMU)*	Spain	University: Cybersecurity and data science
*Novotec Consultores S*.*A*. *(NOVOTEC)*	Spain	Standardisation, quality assessment
**List of internal experts (VALKYRIES expert panel)**		
*Indra Sistemas S*.*A*. *(INDRA)*	Spain	Consulting
*International Security and Emergency Management Institute (ISEMI)*	Slovakia	Emergency management
*Scuola Superiore Sant’Anna (SSSA)*	Italy	University: law school
*Blockchain2050 BV (BC2050)*	Netherlands	Cybersecurity
*Bulgarian Defense Institute (BDI)*	Bulgaria	Military, ciberdefense
*Bulgarian Red Cross (BRC)*	Bulgaria	Emergency coordination
*Center of Security Studies (KEMEA)*	Greece	National security
*Hospital do Espírito Santo de Évora EPE (HESE)*	Portugal	Hospital
*Aratos NTOT NET LTD (ARATOS)*	Greece	Technological services
*University of South-Eastern Norway (USN)*	Norway	University: maritime research
*Azienda Regionale Emergenza Urgenza (AREU)*	Italy	Emergency coordination
*Hellenic Rescue Team (HRT)*	Greece	Emergency coordination
*Particle summary (PARTICLE)*	Portugal	Technological services
*Servicio Madrileño de Salud (SERMAS)* *	Spain	Emergency coordination
*Tassica emergency training & research S*.*A*. (TASSICA)*	Spain	Emergency coordination
*University of Murcia (UMU)* *	Spain	University: Cybersecurity and data science
*Novotec Consultores S*.*A*. (NOVOTEC)*	Spain	Standardisation, quality assessment
**External experts**		
*Emergency Coordination Centre 112 from Extremadura*	Spain	Emergency coordination
*Civil Protection General Direction*. *Extremadura council*	Spain	Civil protection
*Emergencies and Civil Protection General Direction*. *Extremadura council*	Spain	Emergency coordination
*Military Emergency Unit*	Spain	Military emergencies
*Provincial Fire Prevention and Extinction Service of Cáceres*	Spain	Regional firefighter coordination
*National Institute of Medical Emergencies (INEM)*	Portugal	National emergency coordination
*Department for detection of hazardous substances and environmental crime*	Slovakia	National emergency coordination
*Bratislava Emergency Medical Service*	Slovakia	Municipal emergency coordination
*Bratislava Fire and Rescue System*	Slovakia	Municipal firefighter coordination
*General Directorate “Fire safety and Protection of Population”*	Bulgaria	National firefighter coordination
*Military medical academy team*	Bulgaria	National military education
*Bulgarian Emergency ambulance services*	Bulgaria	National emergency coordination
*Bulgarian armed forces*	Bulgaria	National military
*Fire Service Command of the Prefecture of Thessaloniki*	Greece	Regional firefighter coordination
*Hellenic National Centre for Emergency Assistance (EKAB)*	Greece	National emergency coordination
*Norwegian Society for Sea Rescue*	Norway	National emergency coordination

* The internal experts belonging to these organisations were people other than the organisations’ investigators.

**Fig 1:**
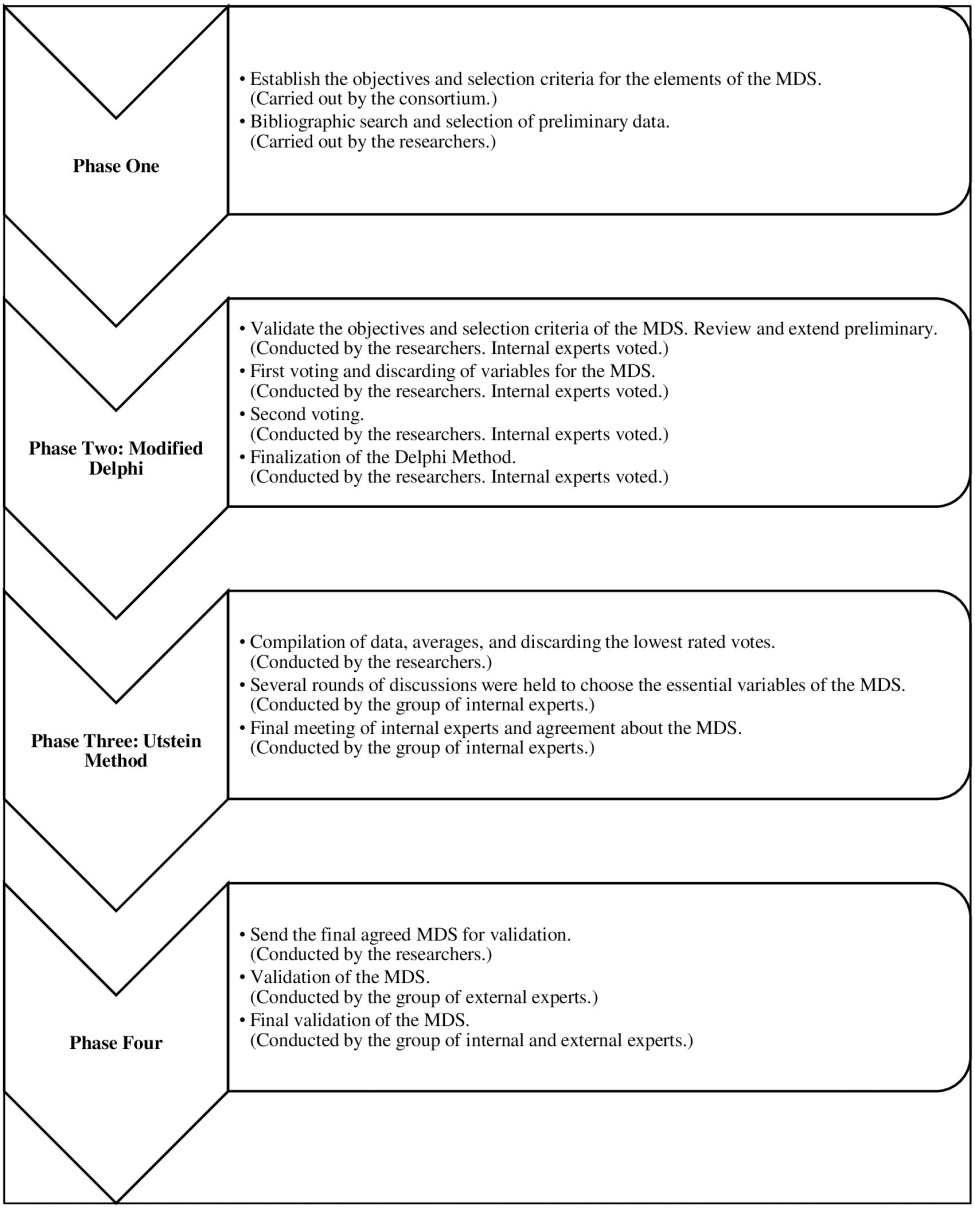
Study development process. *MDS: minimum data set.

The consensus process in four phases described below (Fig 1).

Phase 1—Logistical and operational framework of the process: In this phase, carried out by the consortium, we determined the shared, essential principles to govern the whole process. They were the minimum requirements to be met by the items selected (being thought to be essential to management of MCIs in cross-border areas; suitability for collection, and onward communication of items without any need to change the protocols and procedures of first responders (regardless of place and country)), qualifications and experience of group of experts.

After that, it was necessary to pre-identify the data. This information comes from previous studies [9, 23, 24] and the information currently collected in emergency management in different countries.

Phase 2 –Modified Delphi. It was led by the study team and the internal experts also participated in this phase. There were several face-to-face and online meetings as described below. In the first round of the Delphi method, the scope and structure developed at earlier stages were validated, the researchers organised the preliminary variables into 6 groups based on the literature consulted. Subsequently, each member of the participating suggested new items that they considered important for review. In the first meetings, the objectives and criteria determined in Phase 1 were validated and new items were added at the request of the internal experts. There were then two rounds of voting. Voting required each expert to score each candidate variable from 1 (the item is not material to management of an MCI) to 10 (the item is material for management of an MCI). In Round 1 of voting, items with no votes in favour were discarded. The objectives of the project were reviewed, and a new vote was taken on the variables.

Phase 3 (Utstein Method), led by the researchers, several rounds of discussions were held to choose the essential variables of the MDS. The internal experts who had assigned each variable a value between 1 (not essential for MDS management) and 10 (essential for MDS management) agreed to eliminate those variables with less than 5 points. As a result, the data were ordered from most to least essential for incident management. The internal experts decided that the data with an average score below five should not be considered as essential and, therefore, should not be part of the MDS. This resulted in a digital CMD agreed upon by all the experts in the project.

In Phase 4, the researchers submitted the CMD to external experts, consisting of disaster management and MCI experts from the institutions and agencies involved in the four major simulations foreseen in the project, for final assessment and validation prior to its use in the simulations.

Finally, as part of our modified Delphi strategy, we determined the degree of agreement among the experts by calculating Cohen’s kappa [25, 26].

To that end, we considered agreement between three or more raters (experts) with three or more possible scores (degrees of agreement). In this case, we used a weighted kappa given the number of categories of degree of agreement equivalent to an ordinal scale. The two types of weighting used were squared weights and Cicchetti weights, based on relative distances between categories [27].

The presentation of values of kappa complemented the presentation of the consensus showing degrees of agreement of 55% and above. Data were analysed using IBM’s SPSS Statistics version 2.0. This study did not require consideration and approval by the Ethics Committee of Research with Medicines.

The present study did not need to be submitted to the evaluation of an Ethics Committee since the VALKYRIES Project did not deal with personal data, only simulated data were applied. The Panel (specific working group to ensure legal-ethical compliance with the EU Regulation, which all consortium members signed up to) was made up of ethical-legal experts, who were involved in both compliance and regulatory and standardization tasks, and a declaration was signed by those responsible for the Panel, showing the commitment to comply with all ethical-legal issues. Within this declaration, the decision of the Panel to develop the work without hiring volunteers, not processing personal data, is stated. The case study scenarios involved first responders to receive feedback on the effectiveness of the interoperable platform through the application of simulated and unreal data. The simulated data were generated directly by the Panel.

With regard to inclusivity in global research additional information regarding the ethical, cultural, and scientific considerations specific to inclusivity in global research is included in the S1 Dataset.

## Results

Six data entities were defined with 31 sub-entities (pre-incident data, incident, vehicles, first responders, casualties, and hospitals) that generated the 127 items in the final MDSs, comprising data essential to incident management, capable of being collected and communicated electronically in real-time to the chain of command and control as shown in Table 2. This table presents each data entity and sub-entity identified, in addition to each data entry selected by the organizations participating in the voting process. Moreover, this table contains the data type associated with each data entry to better understand the nature of the information displayed. Furthermore, the table presents one or more examples per data entry to ease the understanding of the content. It is relevant to note that these examples are not agreed values and merely serve as an orientation for interpretation. Finally, Table 2 presents the degree of agreement between voting organizations.

**Table 2:** Presentation and classification of data: Data entities, subentities and items.

DATA ENTITY	DATA SUBENTITY	DATA	DATA TYPE	EXAMPLES	DEGREE OF AGREEMENT
**Pre-event setting**	*HEALTHCARE SYSTEM (INCLUDING CAPACITIES)*	EMS SYSTEM	TEXT	MEDICAL SERVICES FROM MADRID	6
		HEALTHCARE FACILITIES (PRIMARY, SECONDARY, TERTIARY AND SPECIALIST CENTERS)	LIST OF TEXT	HOSPITAL, EMERGENCY CENTER	6
		CRITERIA FOR ACTIVATION OF THE DISASTER MEDICAL MANAGEMENT PLAN (DMMP)	TEXT	FIRE, EARTHQUAKE	6
**Incident MDS**	*CHARACTERIZATION*	INCIDENT ID	NUMBER	1545	10
		DATE OF DECLARATION	DATE	12/02/2022	10
		TIME OF DECLARATION	DATE	12/02/2022	10
		TYPE	NUMBER	5	10
		LOCATION	COORDINATE	41.40338, 2.17403	10
		SEVERITY	TEXT	EXTREME, SEVERE, MODERATE, MINOR, UNKNOWN	6
		STATUS	TEXT	DISPATCHED, ON THE WAY, ON SITE, RESOLVED	6
	*DAMAGE*	ESTIMATED NUMBER OF VICTIMS	NUMBER	60	8
	*HAZARDS (1*..*n)*	ACTIVE HAZARD (Y/N)	BOOLEAN	YES	7.5
		HAZARD LOCATION	COORDINATE	41.40338, 2.17403	6
		HAZARD TYPE	TEXT	CHEMICAL	6
	*FIRST RESPONDERS*	RESCUERS ON SCENE Y/N	BOOLEAN	YES	6
		FIREFIGHTERS ON SCENE Y/N	BOOLEAN	NO	6
		SECURITY FORCES ON SCENE Y/N	BOOLEAN	NO	6
		EMS ON SCENE Y/N	BOOLEAN	YES	6
		OTHER FIRST RESPONDERS ON SCENE	BOOLEAN	NO	6
	*APPROACH (1*..*n)*	LABEL	TEXT	**???**	6
		LOCATION	COORDINATE	41.40338, 2.17403	6
	*ZONATION*	ZERO ZONE LOCATION	COORDINATE	41.40338, 2.17403	6
	*FACILITIES (1*..*n)*	LOCATION	COORDINATE	41.40338, 2.17403	6
		TYPE	TEXT	INCIDENT COMMAND POST, TRIAGE AREA	6
**Vehicle MDS**	*IDENTIFICATION*	VEHICLE ID	NUMBER	42	10
		COUNTRY	TEXT	SPAIN	5
		AGENCY	TEXT	MEDICAL SERVICES FROM MADRID	10
		CODE	NUMBER	87	10
		PLATE	TEXT	12456F	7.5
	*CHARACTERIZATION*	TYPE	TEXT	PASSENGER, LOGISTIC	5
		FUNCTION	TEXT	RESCUE, RECONNAISANCE	7.5
	*POSITIONING*	LOCATION	COORDINATE	41.40338, 2.17403	7.5
		RELATED TO INCIDENT AREA	TEXT	ON THE WAY, ON THE SCENE	10
		RELATED TO ZONATION	TEXT	ZERO ZONE, TRIAGE AREA	5
	*STATUS*	STATUS	TEXT	INOPERATIVE, AVAILABLE	10
	*AMBULANCE (STATIC)*	LIFE SUPPORT CAPACITY	TEXT	BASIC, ADVANCED	5
	*AMBULANCE ASSIGNMENT*	VICTIMS ID	NUMBER	34	7.5
		DESTINATION HOSPITAL ASSIGNED	TEXT	LA PAZ HOSPITAL, VALENCIA	7.5
**First Responder MDS**	*IDENTIFICATION*	RESPONDER ID	NUMBER	43	8
		AGENCY	TEXT	MEDICAL SERVICES FROM MADRID	10
		CODE	NUMBER	14	8
		NAME	TEXT	DOE	5
		SURNAME	TEXT	JHON	5
	*CHARACTERIZATION*	FUNCTION	TEXT	SEARCH, RESCUE	8
		ROLE (C&C)	TEXT	INCIDENT COMANDER, TRIAGE CHIEF	5
	*POSITIONING*	LOCATION	COORDINATE	41.40338, 2.17403	6
		RELATED TO ZONATION	TEXT	MEDICAL AREA, RESCUE AREA	8
	*STATUS*	STATUS	TEXT	INOPERATIVE, AVAILABLE	6
**Victim MDS**	*IDENTIFICATION*	VICTIM ID	NUMBER	11	10
		CODE (RELATED TO INCIDENT)	NUMBER	4	10
		NATIONAL ID	TEXT	12345678J	5
		NAME	TEXT	DOE	5
		SURNAME	TEXT	JOHN	5
	*CHARACTERIZATION*	AGE	NUMBER	24	5
		RANGE OF AGE	INTERVAL OF NUMBER	20–30	10
		GENDER	TEXT	MALE, FEMALE	10
		PREGNANT Y/N	BOOLEAN	YES	7.5
		COUNTRY OF ORIGIN	TEXT	GERMANY, THAILAND	5
		NATIONALITY	TEXT	INDIAN, FRENCH	5
		NATIVE LANGUAGE	TEXT	ENGLISH, SPANISH	5
	*TYPE*	TYPE OF VICTIM	TEXT	DEAD, INJUREND, UNINJURED	7.5
	*CAUTIONS*	ALLERGIES	LIST OF TEXT	PENICILIN, IBUPROFEN	5
		CHRONIC DISEASES	LIST OF TEXT	ASTHMA, ALZHEIMER	5
	*PERSON OF CONTACT (1*,..*n)*	PoC DATA	LIST OF TEXT	JOHN DOE, 90 BEDFORD STREET, NEW YORK	7.5
	*POSITIONING*	LOCATION	COORDINATE	41.40338, 2.17403	7.5
		RELATED TO ZONATION	STRING	MEDICAL AREA, STAGING AREA	10
	*INJURED (Store historical)*	STATUS RELATED TO INCIDENT	STRING	WAITING FOR TRIAGE, IN HOSPITAL	7.5
		STATUS RELATED TO HAZARD	TEXT	TRAPPED, CONFINED, FREE	7.5
		ASSISTANCE TRIAGE	TEXT	URGENT, DELAYED	7.5
		BLEEDING CONTROL	TEXT	NOT NECESSARY, SUCCESSFUL CONTROL	7.5
		AIRWAY (Multiple choice)	TEXT	SUPRAGLOTIC DEVICE, TRACHEAL INTUBATION	7.5
		VENTILATION (Multiple choice)	TEXT	OXYGEN SUPPLY, INVASIVE VENTILATORY SUPPORT	7.5
		HEMODYNAMIC STATUS	TEXT	STABLE, UNSTABLE	7.5
		UNDER INTRAVENOUS FLUIDS Y/N	BOOLEAN	NO	7.5
		INJURIES NATURE	TEXT	PSICHOLOGYCAL, INTOXICATION	7.5
		INJURIES LOCATION	TEXT	NECK, EYES	7.5
		INJURIES SEVERITY	TEXT	MILD, MODERATE, SEVERE	5
		EVACUATION TRIAGE	TEXT	IMMEDIATE, URGENT, DELAYED	10
**Hospital MDS**	*IDENTIFICATION*	HOSPITAL ID	NUMBER	25	
		HOSPITAL NAME	TEXT	LA PAZ HOSPITAL, VALENCIA	7.5
		AGENCY		FRENCH PUBLICH HEALTH CARE SYSTEM	10
		COUNTRY	TEXT	ITALY	7.5
		CITY	TEXT	LONDON	10
		ADDRESS	TEXT	90 BEDFORD STREET, NEW YORK	10
		HOSPITAL LOCATION	TEXT	SPAIN, FRANCE	10
	*GENERAL CONTACT*	GENERAL PHONE NUMBER	NUMBER	1234567789	10
		ED PHONE NUMBER (Emergency Department)	NUMBER	1234567789	8
		ICU PHONE NUMBER	NUMBER	1234567789	7.5
	*HOSPITAL INCIDENT MANAGER*	HOSPITAL INCIDENT MANAGER CATEGORY	TEXT	MEDICAL COORDINATOR	5
	*STATIC DATA*	TOTAL ADULT EMERGENCY DEPARTMENT POSTS	NUMBER	3	5
		TOTAL ADULT ICU POSTS	NUMBER	40	5
		TOTAL ADULT BURN UNIT BEDS	NUMBER	14	7.5
		TOTAL PEDIATRIC EMERGENCY DEPARTMENT POSTS	NUMBER	5	5
		TOTAL PEDIATRIC ICU POSTS	NUMBER	20	5
		TOTAL PEDIATRIC BURN UNIT BEDS	NUMBER	9	7.5
		TOTAL GENERAL SURGERY OP ROOMS NUMBER	NUMBER	7	5
		TOTAL PEDIATRIC SURGERY OP ROOMS NUMBER	NUMBER	4	5
		TOTAL CT NUMBER	NUMBER	3	5
	*DYNAMIC DATA*	AVAILABLE ADULT EMERGENCY DEPARTMENT POSTS	NUMBER	1	5
		AVAILABLE ADULT ICU POSTS	NUMBER	21	7.5
		AVAILABLE ADULT BURN UNIT BEDS	NUMBER	6	10
		AVAILABLE PEDIATRIC HOSPITAL BEDS	NUMBER	33	7.5
		AVAILABLE PEDIATRIC EMERGENCY DEPARTMENT POSTS	NUMBER	2	5
		AVAILABLE PEDIATRIC ICU POSTS	NUMBER	12	7.5
		AVAILABLE PEDIATRIC BURN UNIT BEDS	NUMBER	3	10
		AVAILABLE OPERATING ROOMS NUMBER	NUMBER	5	7.5
		AVAILABLE ORTHOPEDIC SURGERY OP ROOMS NUMBER	NUMBER	1	5
		AVAILABLE GENERAL SURGERY OP ROOMS NUMBER	NUMBER	4	5
		AVAILABLE NEUROSURGERY OP ROOMS NUMBER	NUMBER	1	5
		AVAILABLE THORACIC SURGERY OP ROOMS NUMBER	NUMBER	2	7.5
		AVAILABLE VASCULAR SURGERY OP ROOMS NUMBER	NUMBER	2	5
		AVAILABLE CARDIAC SURGERY OP ROOMS NUMBER	NUMBER	1	10
		AVAILABLE PLASTIC SURGERY OP ROOMS NUMBER	NUMBER	3	10
		AVAILABLE PEDIATRIC SURGERY OP ROOMS NUMBER	NUMBER	4	10
		AVAILABLE OBSTETRIC OP ROOMS NUMBER	NUMBER	2	10
		AVAILABLE CT NUMBER (Computer Tomography)	NUMBER	2	10
		AVAILABLE MRI NUMBER (Magnetic Resonance)	NUMBER	3	10
		AVAILABLE DIALYSIS POSTS NUMBER	NUMBER	5	10
		AVAILABLE ISOLATION ROOMS NUMBER	NUMBER	3	10
		AVAILABLE HYPERBARIC CHAMBERS NUMBER	NUMBER	1	10
	*TASK ASSIGNED*	TASK ID	NUMBER	4	10
		INCIDENT ID	NUMBER	5	10
		VEHICLE ID	NUMBER	42	7.5
		HOSPITAL ID	NUMBER	25	10
		VICTIMS ASSIGNED	LIST OF NUMBER	11, 14, 21	10

*MDS: minimum data set

Therefore, the data obtained is presented as follows:

### 1. Pre-event setting MDS

The data related to the pre-event configuration collected those related to population groups, health systems, critical infrastructures, climatological factors, and resource management.

The collection of previous data was essential for efficient management and based on risk prevention. For this reason, the experts participating in the selection of the data relied on previous studies and relevant information analysed to arrive at the proposed data.

Here the sub-entity *healthcare system (including capacities)* was generated with 3 items that obtained a level of agreement of 6/10 for all. Their name, classification and order are shown in Table 2.

### 2. Incident MDS

The MDS of the incident mainly collected the characterization of the incident, damages, risks, vulnerability, resources of first responders in the event, access routes, zoning, and facilities. This information was collected in any of the scenarios. The complexity was linked to the nature and characteristics of said incident.

Here the sub-entities *characterization*, *damage*, *hazards*, *first responders*, *approach*, *zonation* and *facilities* were generated with 21 items that obtained a degree of agreement of 10/10 (from “incident ID”, date of declaration, time of declaration type “location "), 8/10 ("estimated number of victims"), 7.5/10 ("active hazards") and 6/10 ("severity", “status hazard location”, “hazard type”, “rescuers on scene”, “firefighters on scene”, “security forces on scene”, “EMS on scene”, “other first responds on scene”, “label location”, “zero zone location”, “location”, “type”).

### 3. Vehicle MDS

The vehicles were collected in a separate section to identify them, characterize them, position them, and know their status. A data set was proposed for ambulance devices, victim parameters in ambulance transport, and ambulance assignment.

Here the sub-entities *identification*, *characterization*, *positioning*, *status*, *ambulance (static) and ambulance assignment* were generated with 14 items that obtained a degree of agreement of 10/10 (“vehicle ID”, “agency”, “code”, “related to incident”, “area status “), 7.5/10 (“plate”, “function”, “location”, “victims ID”, “destination hospital assigned”) and 5/10 (“country”, “type”, “related to zonation”, “life support capacity”).

### 4. First responder MDS

The first responders required a set of data for identification, characterization, positioning, and status.

Here the sub-entities *identification*, *characterization*, *positioning*, and *status* were generated with 10 items that obtained a degree of agreement of 10/10 (“agency)”, 8/10 (“respond ID”, “code”, “function”, “related to zonation”), 6/10 (“location”, “status”) and 5/10 (“name”, “surname”, “role”).

### 5. Victim MDS

Regarding the victims, data related to their identification, characterization, type, precautions, contact person, positioning, injuries, health monitoring data and other information related to the person were grouped.

Here the sub-entities *identification*, *characterization*, *type*, *cautions*, *person of contact*, *positioning* and *injured (store historical)* were generated with 18 items that obtained a degree of agreement of 10/10 (“victim ID”, “code (related to incident)”, “range of age”, “gender”, “related to zonation”, “evacuation triage”), 7.5/10 (“pregnant”, “type of victim”, “poc data”, “location”, “status related to incident”, “status related to hazard”, “assistance triage”, “bleeding control”, “airway”, “ventilation”, “hemodynamic status”, “under intravenous fluids”, “injuries nature”, “injuries location”) and from 5/10 “(national ID”, “name”, “surname”, “age”, “country of origin”, “nationality”, “native language”, “allergies”, “chronic diseases”, “injuries severity”).

### 6. Hospital MDS

Finally, hospital-related information, such as identification, contact route, hospital incident manager, statistical data and dynamic data needed for resource and incident management were specified.

Here the sub-entities *identification*, *general contact*, *hospital incident manager*, *static data*, *dynamic data* and *task assigned* were generated with 46 items that obtained a degree of agreement of 10/10 (“agency”, “city”, “address”, “hospital location”, “general phone number”, “available adult burn unit beds”, “available pediatric burn unit beds”, “available cardiac surgery op rooms number”, “available plastic surgery op rooms number”, “available pediatric surgery op rooms number”, “available obstetric op rooms number”, “available CT number (computer tomography)”, “available MRI number (magnetic resonance)”, “available dialysis posts number”, “available isolation rooms number”, “available hyperbaric chambers number”, “task ID”, “incident ID”, “hospital ID”, “victims assigned”), 7.5/10 (“hospital name, country”, “ICU phone number”, “total adult BURN unit beds”, “total pediatric burn unit beds”, “available adult ICU posts”, “available pediatric hospital beds”, “available pediatric ICU posts”, “available operating rooms number”, “available thoracic surgery op rooms number”, “vehicle ID”) 8/10 (“ED phone number (emergency department)”) and from 5/10 (“hospital incident manager category”, “total adult emergency department posts”, “total adult ICU post”, “total pediatric emergency department posts”, “total pediatric ICU posts”, “total general surgery op rooms number”, “total pediatric surgery op rooms number”, “total CT number”, “available adult emergency department posts”, “available pediatric emergency department posts”, “available orthopedic surgery op rooms number”, “available general surgery op rooms number”, “available neurosurgery op rooms number”, “available vascular surgery op rooms number”).

The 127 items finally agreed for inclusion in the MDS can be readily recorded in real time by first responders to an MCI thanks to a system for the collection and communication of digital data called SIGRUN, developed under Project VALKYRIES. In fact, the agreed MDS was tested in the four simulations run by the EU’s Project Valkyries. The medical emergency response services of the eight countries involved in the simulations at four cross-border and cross-sectorial scenarios (Spain-Portugal, Slovenia-Italy, Bulgaria-Greece, Norway-Netherlands) were able to gather and communicate the MDS items to the chain of command and control for each incident in real time. They were able to confirm the effectiveness of this new harmonized tools in different highly complex scenarios, such as: forest fires; earthquake; chemical, biological, radiological and nuclear (CBRN) and offshore disasters. The results of those simulations will be published in the coming months.

The results with the highest level of consensus in our study are for the MDS for the category ‘Incident’ with overall kappa of 0.7401 (95% CI 0.1265–0.5812, p 0.000), a good level of consensus in the Landis and Koch model [25]. The items with the greatest degree of consensus (10 out of 10) were those relating to location, type of incident, time and identification of the incident.

With moderate values (kappa of 0.4–0.6) in the Landis and Koch model, we find the description of the situation prior to the incident, with overall kappa of 0.44 (95% CI 0.0114–0.2531, p 0.0093) and ‘first responders’ with overall kappa of 0.4318 (95% CI 0.0599–0.5928, p 0.0000). The degree of agreement on the items concerning pre-incident information was similar (6 out of 10). Conversely, the disposition, function and type of first responders garnered the highest level of agreement, in excess of eight out of ten.

The categories that had low levels of agreement (kappa 0.2–0.4) were vehicles with kappa of 0.2696 (95% CI 0.0780–0.2482, p 0.0000); casualties, with kappa 0.2320 (95% CI 0.0261–0.1393, p 0.0006); and hospitals, with kappa of 0.3241 (95% CI 0.1076–0.1500, p 0.0000). However, the degree of agreement was maximal for some items, including the personal details of patients and their patient reference numbers to ensure maximum traceability; patient age band and gender; location; and triage colour code. All results for degrees of agreement are shown in the Table 3.

**Table 3:** Levels of agreement between participating agencies.

Data Entity	Kappa	Z Statistic	95% CI	p-value
***1*. *Pre-event setting***				
**Categories of Degrees of Agreement**				
**Not Applicable**	0.0308	0.4128	-0.1132 0.1736	0.6797
**2**	-0.0465	-0.6240	-0.0928 0.0003	0.5326
**4**	0.1346	1.8061	0.0390 0.2300	0.0709
**6**	0.4444	5.9628	0.3755 0.5132	0.0000
**Overall Kappa**	**0.4444**	**2.6009**	**0.0114 0.2531**	**0.0093**
***2*. *Incident MDS***				
**Categories of Degrees of Agreement**				
**Not Applicable**	-0.0756	-1.0960	-0.1951 0.0435	0.2731
**6**	0.1667	2.4152	-0.0202 0.3526	0.0157
**7.5**	0.4853	7.0326	-17.9661 19.8414	0.0000
**8**	0.7401	10.7251	-17.2165 19.5776	0.0000
**10**	1.0000	14.4914	1.0000 1.0000	0.0000
**Overall Kappa**	**0.7401**	**8.1186**	**0.1265 0.5812**	**0.0000**
***3*. *Vehicle MDS***				
**Categories of Degrees of Agreement**				
**Did not vote**	-0.1809	-2.6215	-0.2069–0.1551	0.0088
**Not Applicable**	-0.0111	-0.1610	-0.1070 0.0838	0.8721
**5**	0.2735	3.9636	-0.0911 0.6320	0.0001
**7.5**	0.2696	3.9064	0.1432 0.3952	0.0001
**10**	0.5302	7.6827	0.3553 0.7035	0.0000
**Overall Kappa**	**0.2696**	**4.5643**	**0.0780 0.2482**	**0.0000**
***4*. *FIRST RESPONDERS MDS***				
**Categories of Degrees of Agreement**				
**Did not vote**	-0.0638	-0.6383	-0.1414 0.0135	0.5233
**Not Applicable**	-0.1409	-1.4087	-0.2563–0.0272	0.1589
**5**	0.1477	1.4773	0.0160 0.2786	0.1396
**6**	0.4318	4.3182	0.3195 0.5432	0.0000
**8**	0.6324	6.3235	0.4745 0.7872	0.0000
**10**	1.0000	10.0000	-16.4235 20.2235	0.0000
**Overall Kappa**	**0.4318**	**6.5389**	**0.0599 0.5928**	**0.0000**
***5*. *Victim MDS***				
**Categories of Degrees of Agreement**				
**Did not vote**	-0.1447	-3.1706	-0.2259–0.0637	0.0015
**Not Applicable**	-0.0722	-1.5824	-0.1240–0.0206	0.1136
**5**	0.1070	2.3437	0.0508 0.1631	0.0191
**7.5**	0.2320	5.0829	0.1428 0.3210	0.0000
**10**	0.4742	10.3894	0.3819 0.5661	0.0000
**Overall Kappa**	**0.2320**	**3.4197**	**0.0261 0.1393**	**0.0006**
***6*. *Hospital MDS***				
**Categories of Degrees of Agreement**				
**Did not vote**	-0.1970	-5.3405	-0.2030–0.1909	0.0000
**Not Applicable**	0.0046	0.1238	-0.0421 0.0512	0.9015
**5**	0.1158	3.1392	0.0732 0.1583	0.0017
**7.5**	0.3241	8.7877	0.2783 0.3699	0.0000
**8**	0.5945	16.1170	-17.7390 19.3117	0.0000
**10**	0.4178	11.3275	0.3360 0.4995	0.0000
**Overall Kappa**	**0.3241**	**0.3241**	**0.1076 0.1500**	**0.0000**

*MDS: minimum data set

## Discussion

Scientific publications on MCI are still very scarce, even more so when it comes to cross-border events involving international organisations. To date, there is no evidence that determines what MDS should be collected or how that data should be collected and managed. Consequently, the principal strength that can be claimed for the method employed is that it achieved consensus in areas where there is no certainty or where evidence on a topic is empirical [9].

In addition, the results advance the principal objective of the project because although there are a number of limited-scope MDSs, there is currently no exhaustive MDS and no analysis across a range of cross-border disaster scenarios, given that information has traditionally been collected from observation or analogue capture. However, this study worked in parallel on the capture of digital data, as will be described in subsequent articles.

One of the problems observed in the management of MCI is variability in the way in which information is gathered, which may in turn lead to divergent results and so hinder comparison and standardization of those results [28]. That is all the more so in cross-border (MCI), such as the simulations that were conducted in an earlier phase of this project.

Another problem in the management of disasters seen in connection with MDSs for casualty information and how to collect that information is that not all the information about a casualty should be shared with all the agencies involved in an MCI. However, some part of that information may be important for command-and-control decision-making [29].

Modified Delphi and subsequent Utstain-style technique allowed a digital MDS to be developed with the involvement of 17 experts from eight organizations and institutions involved in the Valkyries project. Experts from eight different countries with extensive experience of emergency and disaster management, IT and cybersecurity, standardisation, etc. validated this MDS, which was subsequently tested in four large-scale simulations in eight European countries.

This tool complies with the Health Disaster Management: Guidelines for Evaluation and Research issued by the Working Group on Quality Assurance in Disaster Management (WADEM) [30] and the tool developed by the Academy for Emergency Management and Disaster Medicine (EMDM Academy) [9] which provides standardised systematic empirical information on disaster medical response to support monitoring for further evaluation, training and research.

The variability across procedures and action protocols of organizations with responsibility for MCI/disaster management and the variability of technical and human resources, with different degrees of training, education and specialisation, can make it difficult to provide an optimal joint response. This further complicates the evaluation, training and investigation of such incidents [31–35]. The digital MDS that has been developed and tested in the four simulations makes possible standardized systematic collection of data that supports the monitoring of many aspects of incident management and so in turn undoubtedly lead to the identification and assessment of areas for improvement, training, guidance and research in this field.

Data collection in the course disaster management has not thus far been regulated [34, 36–38]. The MDS in this study is based on current scientific evidence and allows for the systematic collection of data in real time, as shown by the collection of data in large-scale simulations involving eight European countries–in a field of research sorely lacking empirical research [28, 39]. The automatic digital collection of data speeds the provision of healthcare, which is one of the most significant issues in the reporting of medical care in such incidents [36, 40, 41].

It is also significant that information about casualties does not always originate from health services. Casualties may receive initial care from other agencies, such as firefighters, police and volunteers and that information is not recorded in support systems, leading to loss of information as a casualty passes from the care of one service into the care of another service. Consequently, the way in which information should be processed is not always the same and applicable laws relating to the collection of sensitive personal data should be followed at all times and the rights of casualties under the General Data Protection Regulation (GDPR) should be given full effect.

The advantage of the tool lies in its neutrality with the way of working of first responders, their action protocols, language, etc. These MDS is put forward as the minimum essential data for optimal MCI management. Another advantage of the digital MDS presented is the possibility of combined collection and digital dissemination in real time to the entire chain of command and control, both in the country of collection and to other countries. For example, in the simulation exercise carried out in May 2023 on the border between Spain and Portugal, it was possible to record all the items digitally in real time and international command across the two countries had information on the number of casualties, their characteristics and the resources involved at all times. The data were stratified and protected so that each responder could only access data related to their specialism.

The healthcare coordinators, like the security services, did not have access to specific medical data for patients, but they were informed in real time of the number and severity (colour) of each Triage station. Conversely, doctors and nurses at the medical stations could access patients’ medical report, but not other non-medical data such as numbers and types of resources known to the incident commander.

We thus eliminated the need to use telephones or radio to communicate the information needed to manage the incident with a significant impact on delays to incident management pending availability of information.

For that reason, in this study the expert consensus reached through Delphi focused on optimizing incident management and minimizing the need to communicate information by voice and maximizing the traceability of casualties and the management of transport, among other data. It was not therefore focused on the medical treatment or management of individual patients. Interventions of this nature are particularly useful when there are issues such as remoteness and challenging geographies, requiring the intervention of teams from different countries, with different languages and ways of working. The scope for error is reduced by a standardized procedure in especially complicated situations such as (MCI), which can reduce the time taken by healthcare staff in treating casualties and so reduce morbimortality and allow investigators to collect and interpret reliable evidence to improve survival and reduce morbidity among patients treated in an MCI.

As can be seen from Table 3, the degree of consensus was as high as possible (10/10) among experts for the basic items that directly affect incident management, such as identification of the incident, its location, type of resources, casualty triage, etc. We should remember that all data was gathered and communicated in real time to the whole chain of command and control, greatly improving the management of the four large simulations run to confirm the effectiveness of this tool. It is also important that it proved possible to collect the data regardless of place and language, with full respect throughout for the procedures, protocols, and laws of each country.

The value for Cohen’s kappa to be taken as showing a good level of agreement varies depending on the problem at hand. In practice, we have used a scale for the interpretation of kappa values from Acceptable as equal to or greater than 0.40 (First Responder) to Excellent as above 0.75 (for Incident) [26].

However, our results (particularly in relation to casualties and hospitals) show that it is possible for there to be a high level of observed agreement and, at the same time, a kappa of close to zero. The apparent paradox of high levels of observed agreement and low kappa values can be explained by the fact that for a given level of observed agreement, the value of kappa depends on the frequency or rarity of the phenomenon under study [27]. Among the solutions proposed for this problem is to state the maximum and minimum values of kappa with the calculated value of kappa. We must also bear in mind that as the number of categories increases, so does the difficulty of correctly classifying the observed items, which generally leads to lower values of kappa [42].

It is now worth noting a peculiarity of this study, namely the use of Kappa to measure the degree of agreement of the evaluations. That this is uncommon can be confirmed from a review of prior similar studies in the same area [43–46] where such calculations were not carried out. In another case the Kendal coefficient was used [47]. The Kendal coefficient is used to measure associations between ratings and is suitable for ordinal rankings (ratings on a scale), which is not the case in this study [48]. As noted above, Kappa is particularly suitable for use with a Delphi-type consensus method.

Studies such as that by Debacker M. et al. [9], involving the development of a standardized model for the management of data in catastrophes, proposed testing such models in the field. That is where the novelty of this study lies, that we have tested the degree of agreement of this gathering of Utstein-style MDS not only in situ but in cross-border MCIs, thus giving a good representation of the operation of the model.

On the basis of the data obtained through Delphi, we propose conducting further projects to address the challenges that we encountered. To assess the potential usability of metadata where data are collected electronically. Analysis should be carried out after the fact with exact response times. A trial should be conducted to determine the format of such information: there are many choices, such as triage colour, age band, numerical values such as age and alphanumeric information such as name and surname(s). In conclusion, to find a solution to the problem of data segmentation.

In terms of limitations, while a major step forward has been made in bringing this method to the field, it is still a simulation. To assess its full potential, the MDS would have to be tested in real-world multi-casualty incidents. A second limitation is testing only in European countries: it should also be tested in areas undergoing development to assess its suitability. The modified approach adopted in this Delphi survey is considered superior to the original approach because it is highly effective and less time consuming. Having an “expert panel” is central to the process of the Delphi technique, although there are no standard criteria for determining expertise. In the current study, the panel of experts comprised people from several countries/cities who are in diverse professions, such as university academics, physicians, and nurses.

## Conclusion

This study documents the development of an MDS through consensus with a high degree of agreement in a group of experts of different nationalities working in different fields. All items in the MDS were digitally collected and communicated in real time to the chain of command and control. This tool has demonstrated its validity in four large cross-border simulations involving more than eight countries and their emergency services using a method that generates proposals for further investigation. The work falls within the framework of proposals made in the Conclusions of the Council of Europe for Disaster Risk Reduction in EU external action (28 November 2022).

## Supporting information

S1 Checklist Inclusivity in global research. (DOCX)

S1 Dataset (XLSX)
