# Supplementary material for: Minimum data set harmonization in the management of cross-border Multi Casualty Incidents. Modified Delphi (VALKYRIES—H2020 project)
Source: PLoS One. 2024 Jul 18;19(7):e0305699. doi: 10.1371/journal.pone.0305699 (PMC11257232; doi:10.1371/journal.pone.0305699)
Supplement: S1 Checklist — (DOCX) [file pone.0305699.s001.docx]

Inclusivity in global research

PLOS’ policy on inclusivity in global research aims to improve transparency in the reporting of research performed outside of researchers’ own country or community and ensures that PLOS publications reporting global research adhere to high standards for research ethics and authorship. Authors of relevant research articles may be asked to complete the questionnaire below, which outlines ethical, cultural, and scientific considerations specific to inclusivity in global research. This questionnaire may be requested when researchers have travelled to a different country to conduct research, if research uses samples collected in another country, research with Indigenous populations or their lands, or if research is on cultural artefacts. Researchers travelling to another country solely to use laboratory equipment will not normally be required to complete the questionnaire. However, the questionnaire can be requested at the journal’s discretion for any submission – if you have been requested to complete this questionnaire by the PLOS journal you submitted to, please do so.

Please complete the questionnaire below and include this as a Supporting Information file with your manuscript. Note that if your paper is accepted for publication, this checklist will be published with your article in the supporting information files. Please ensure that you reference the checklist in the main body of your manuscript. We suggest adding a subsection ‘Inclusivity in global research’ to your Methods section and adding the following sentence: “Additional information regarding the ethical, cultural, and scientific considerations specific to inclusivity in global research is included in the Supporting Information (SX Checklist)”

The questions have been designed to be applicable to a wide range of study types, and there are subsections for both human subjects research and non-human subjects research. If any of the questions are not relevant to your research please mark them as “N/A” as appropriate.

**Ethical considerations, permits and authorship**

*This section is applicable to all research types.*

Provide details as to who granted permissions and/or consent for the study to take place in the Methods section of your manuscript. This should include the names of **all** ethics boards, governmental organizations, community leaders or other bodies that provided approval for the study. If individuals provided approval refer to these people by their role or title but do not list their name(s).

Reported on page number: **13**.

VALKYRIES ethical legal profiles are addressed under T1.5 that is supposed to provide a continuous assessment of the technical activities undertaken during the life cycle of the research. To this end, the Consortium is composed of ethical-legal experts, who intervene both in compliance and regulatory and standardisation tasks. This deliverable, introduced by the EU Commission, will explain how the Consortium decided to not engage volunteers and not processing personal data. Further details are submitted under D8.3, specifically addressing case-studies activities.

Supporting documents are attached to this manuscript. On the other hand, the project was presented to the authorities in charge of disaster management and the first responders in the border areas where the four simulations were carried out, who agreed to participate in the simulations altruistically and without receiving any type of compensation, with the aim of improving the training of first responders in this type of disaster. The experts from each organisation were aware of the variables to be collected in the project and gave their approval to create the final MDS, although they did not participate directly in this study or in the Valkyries project.

If there were any deviations from the study protocol after approval was obtained please provide details of these changes in the Methods section of your manuscript.
Did this study involve local collaborators that are residents of the country where the research was conducted or members of the community studied? If you do not have any authors from said communities, please provide an explanation for this below.

The participation of disaster management authorities and first responders was altruistic and without compensation. Their participation was accepted to encourage their training in this type of disaster and the possibility of working together with other first responders in the neighboring country. In no case were they involved in the planning, organization or data analysis of this study or the Valkyries project.

Reported on page number: **N/A**

**N/A**

Everyone listed as an author should meet PLOS’ criteria for authorship and all individuals who meet these criteria should be included in the author byline, rather than the acknowledgements. For further information please see the journal’s Authorship Policy.

**Human subjects research (e.g. health research, medical research, cross-cultural psychology)**

Did you obtain written informed consent from a representative of the local community or region before the research took place? How did you establish who speaks for the community? Details of written informed consent obtained from study participants should be reported separately in the Methods section of your manuscript.

The present study did not need to be submitted to the evaluation of an Ethics Committee since the VALKYRIES Project did not deal with personal data, only simulated data were applied. The Consortium was made up of ethical-legal experts, who were involved in both compliance and regulatory and standardization tasks, and a declaration was signed by those responsible for the Consortium, showing the commitment to comply with all ethical-legal issues. Within this declaration, the decision of the Consortium to develop the work without hiring volunteers, not processing personal data, is stated. The case study scenarios involved first responders to receive feedback on the effectiveness of the interoperable platform through the application of simulated and unreal data. The simulated data were generated directly by the Consortium. Two annexes containing the commitments made are attached. The first responders participating in each exercise were organized in the normal way by the authorities in charge of disaster management in each country, who obtained the necessary permits for the execution of the drills as is customary in the normal drills of each organization.

How did members of the local community provide input on the aims of the research investigation, its methodology, and its anticipated outcome(s)?

In phase 4 of the process of reaching consensus to create the MDS, the database was presented to the disaster management authorities in each country and to the experts assigned by them for approval prior to conducting the exercises. After each exercise, meetings were held with the chain of command and control and first responders to get their feedback on the exercise and the tool developed for the digital collection of the MDS. In addition, a survey on the usefulness and usability of the tool was carried out, which is currently being analysed and we hope to publish it in the coming months.

When engaging with the local community, how did you ensure that the informed consent documents and other materials could be understood by local stakeholders?

Prior to each drill, those responsible for each drill contacted the local authorities to present the project and invite them to participate in the drills, making it clear that their participation would be altruistic and without any type of compensation. All the organisations agreed to participate in the drills with the aim of encouraging training and joint work in this type of disaster. Subsequently, several meetings were held with the authorities and first responders to explain the work logistics and clarify any doubts.

Will the findings of the research be made available in an understandable format to stakeholders in the community where the study was conducted (e.g. via a presentation, summary report, copies of publications, etc.)? Please provide details of how this will be achieved.

The preliminary results of the Valkyries project have been presented to the disaster management authorities of the border areas and the first responders where the exercises were conducted. There is a strong commitment to communicate the final data and publications to them, taking into account that the project was very well evaluated by the participants of the exercises and although they are preliminary results, the vast majority found the tool useful or very useful for their daily work.

**Non-human subjects research using specimens/ animals collected as part of the study, or those housed in archival collections. Examples include archaeology, paleontology, botany and zoology.**

Did the permission you obtained from a local authority to perform the study include an agreement on access to outputs and benefit sharing? This may include procedures to enable fair distribution of the benefits and resources arising from the research performed. Please include any details of Prior Informed Consent and Benefit Sharing Agreements obtained. These may be required by field-specific regulations, for example the Convention on Biological Diversity (CBD) and the associated Nagoya Protocol.

**N/A**

If the material used in your study was imported, please A) provide the year it was imported and B) indicate whether permits were obtained to import/export the materials used, C) provide details of any permits obtained. If this information is not available, please indicate this.

**N/A**

If you used archival specimens, please state how the material used in your study was acquired by the institute it is held in and provide details of any permits obtained for the original excavations/ sample collection. If this information is not available, please indicate this.

**N/A**

How was the potential cultural significance of the materials collected in your study to local communities considered in your research design? Were Indigenous peoples and/or local researchers and institutions involved with archaeological excavations / collection of specimens? If so, please provide a description of their involvement.

**N/A**

If your manuscript includes photographs of human remains please indicate whether authors obtained permission from descendants or affiliated cultural communities to do so.

**N/A**
